# Survey describing the perspectives and practices of Australian veterinarians to pain management in horses

**DOI:** 10.1111/avj.70059

**Published:** 2026-02-05

**Authors:** A Whitelock, W Goodwin, L Dryburgh, PP Mshelbwala, L Rae, L Marwedel, T Lok, K Kemp, AJ Stewart

**Affiliations:** ^1^ School of Veterinary Sciences The University of Queensland Gatton Queensland 4343 Australia; ^2^ Boehringer Ingelheim Animal Health Australia 78 Waterloo Rd North Ryde New South Wales 2113 Australia; ^3^ Department of Primary Industries and Regional Development 105 Prince St Orange New South Wales 2800 Australia; ^4^ Randwick Equine Specialists Horsley Park New South Wales 2175 Australia

**Keywords:** analgesia, equine, survey, veterinary

## Abstract

**Objective:**

To describe Australian practices and attitudes regarding equine analgesia.

**Study Design:**

Cross‐sectional anonymous, voluntary survey of Australian veterinarians treating equine patients.

**Methods:**

Australian veterinarians in equine or mixed practices completed a six‐section, 60‐question survey between November 2019 to August 2020. Information was gathered on demographics, analgesia prescription, pain assessment and attitudes surrounding specific analgesics. Respondents assigned a pain score ranging from 0 to 10 for various conditions; these were averaged to give each respondent an “average pain score”.

**Results:**

Data from 153 respondents were included for analysis. The majority of respondents were female (68%). There was no obvious effect of gender on practice type, with approximately half of respondents working in equine exclusive (50.3%) or mixed (49.7%) practice irrespective of gender. Butorphanol was the most frequently used opioid, with 25.5% and 39.2% of respondents using it “every day” and “a few times a week”, respectively. The use of nonsteroidal anti‐inflammatory drugs (NSAIDs) was diverse, with phenylbutazone, flunixin and meloxicam being the most administered. Formal pain scales were infrequently used, with more than 35% of respondents reporting no pain scale being utilised commonly. In response to various clinical scenarios, veterinarians graduating less than 10 years ago were more likely to assign high average pain scores compared to respondents graduating more than 10 years ago (78.0% vs. 66.0%, respectively). Female veterinarians were more likely to assign high average pain severity scores than males (73.0% vs. 60.0%, respectively). However, in the multivariable analysis, none of the predictors were found to be statistically significant.

**Conclusions:**

This survey demonstrates that demographic factors influence the attitudes of Australian equine veterinarians regarding pain and there may be opportunities to educate and optimise pain assessment and protocol choices.

The recognition and treatment of pain improves disease outcomes and is an ethical obligation in equine veterinary practice. Appropriate pain management in horses has been shown to improve animal welfare, decrease length of hospitalisation and reduce weight loss.^1^, ^2^, ^3^ Pain is a complex, multifaceted process involving both the peripheral and central nervous system.^4^ Pain, as supported by the International Association for the Study of Pain, refers to an unpleasant sensory and emotional experience associated with, or resembling that associated with, actual or potential tissue damage.^5^ This sensory experience results in a change in physiology and behaviour to avoid tissue damage and facilitate recovery. It is important to acknowledge that the nonverbal nature of veterinary species does not negate the possibility of pain. Nonfunctional pain occurs when there is an inappropriate duration or intensity of pain which is not alleviated through behavioural and physiological mechanisms.^6^ This definition incorporates the process of both acute, protective pain and chronic, maladaptive pain.

Considerable progress has been made regarding equine pain scoring, interventions and prevention.^7^, ^8^, ^9^, ^10^, ^11^, ^12^, ^13^, ^14^, ^15^ Despite this progress, much uncertainty and a lack of compelling evidence exist relating to the treatment of pain in horses compared with people, dogs and cats.^8^, ^16^, ^17^ These challenges are centred around the accurate, reliable recognition of pain in a nonverbal prey species and a scarcity of theoretical and clinical evidence regarding useful analgesics.^8^, ^13^, ^18^, ^19^ Recent literature has focused on the development of equine pain scoring systems to allow standardised assessment of pain and therefore allow titration and recognition of adequate analgesia. Proposed equine pain scoring measures have been plagued by poor inter‐observer agreement when using Visual Analogue Scales (VAS)^20^ and reliance on anatomic pathology specific measures (e.g., limb palpation, lameness scoring, flank kicking).^10^, ^12^, ^21^ Use of facial pain scales appears promising due to their high inter‐observer agreement, ease of training and nonpathology specific nature.^10^, ^11^, ^13^, ^22^

The evaluation of current techniques and attitudes towards the recognition and treatment of pain has been shown to improve clinical outcomes in human medicine.^23^, ^24^, ^25^, ^26^ No information regarding current practices relating to the recognition, treatment or attitudes towards pain in horses in Australia exists. The purpose of this study is to describe the perspectives, prescribing practices and recognition of pain in horses in Australian veterinary practice.

## Material and methods

Data were collected using an online questionnaire conducted through Qualtrics XM from November 2019 to August 2020 and consisted of 60 questions. The survey was open to all equine veterinarians in Australia and promoted through advertisements in Boehringer Ingelheim company newsletters, email, promotional materials and in‐person visits to veterinary clinics (Appendix A).

The questionnaire consisted of six sections. Section 1 collected demographic information (gender, age, year of graduation, proportion of equine patients, type of practice) and information relating to how frequently analgesics were administered to horses. Section 2 gathered information on the use of pain scoring methods (Composite Pain Scale, Equine Acute Abdominal Pain Scale (EAAPS), Equine Utrecht University Scale for Composite Pain Assessment (EQUUS COMPASS), UNESP‐Botucatu Multidimensional Pain Scale, Facial Expression Scale and the American Association of Equine Practitioners (AAEP) lameness scale^10^, ^11^, ^12^, ^17^) and if utilised, who is responsible for pain scoring horses. Section 3 respondents were asked to describe their use of nonsteroidal anti‐inflammatory drugs (NSAIDs) and opioids, whether they were routinely used in the pre‐, intra‐ or postoperative period and what factors influenced their selection of specific NSAIDs and opioids. Information was also gathered regarding patient monitoring when NSAIDs were used. Section 4, respondents were asked to describe when analgesics (NSAIDs, opioids, local anaesthetics, alpha‐2 agonists or “other”) were administered to patients with specific clinical conditions and for how long these therapeutics were administered when used postoperatively. Specific questions regarding NSAID and opioid use were included as it was felt these medications were likely to be the most commonly utilised medications in equine practice. Specific clinical scenarios included large routine dental procedure, Caslick's vulvoplasty, laceration repair, sequestrum debridement, medical management of corneal ulceration, castration and severe acute bilateral forelimb laminitis. Data for colon volvulus exploratory laparotomy, “tie‐back” (laryngoplasty) surgery, surgical repair of deep corneal ulceration, condylar third metacarpal fracture, bilateral fetlock arthroscopy were also collected however is not presented within this report due to lower response rates. Section 5 required respondents to estimate pain severity on a 0–10 point scale for a selection of conditions in Section 4. Section 6 investigated NSAID use and rationale in osteoarthritis. Data from portions of the survey sections are combined and presented in the results.

### Statistical analysis

Descriptive statistics were used to analyse the sociodemographic data and prescribing habits. Each respondent was given an “average pain score” based on the pain scores they provided for the conditions in Section 5. Based on the average pain scores respondents were grouped in those that generally scored a low pain score (0–5 average pain score) or those that generally scored a high pain score (6–10 average pain score) to allow for further data analysis. A univariable logistic regression using the open‐source software R (version 3.6.2) in RStudio was then constructed assessing all variables' association with respondents' pain score grouping. All variables with a P‐value of 0.20 or lower in the univariable analysis were considered for inclusion in a multivariable model. A backward stepwise approach was applied to develop a main effects model, using a significance level of 0.1 and retained confounders irrespective of their statistical significance. Confounding was examined by assessing changes in the coefficients of the remaining significant variables. If a variable's coefficient changed by more than 20%, the removed variable was identified as a confounder and reintroduced into the model. A P‐value of less than 0.05 was regarded as statistically significant. Finally, after completing the multivariable model, the model's fit was assessed using the Akaike information criterion (AIC).

## Results

### Demographics

The questionnaire had 221 respondents; of these, 153 were included for data analysis. Exclusions were due to failure to answer more than 25% of the questionnaire (58 respondents) or answering “none” to weekly equine patients examined by the practitioner (10 respondents). The demographic information of respondents is described in Table 1. The majority of respondents were female (68%), with 57% of the female respondents having been graduated for 10 years or less. Male respondents tended to have been graduated longer, with 60% of male respondents having graduated more than 10 years ago. There was no effect of gender on practice type, with approximately half of respondents working in either equine exclusive practice (50.3%) or mixed practice (49.7%) irrespective of gender. The majority of respondents were from eastern Australia, with 87.8% of respondents working in Queensland, New South Wales or Victoria.

**Table 1:** Demographics of 153 Australian veterinarian respondents

Demographics of 153 Australian veterinarian respondents to an equine pain survey (%)																	
	Years since graduation						Practice type		University of Graduation								Total
	0–2 years	3–5 years	6–10 years	11–20 years	21+ years	Not answered	Equine	Mixed	UQ	USYD	UM	CSU	MU	JCU	AU	Other	
Female	5.2	14.4	19.0	15.0	6.5	7.8	34.0	34.0	15.7	11.1	8.5	11.1	7.2	1.3	3.3	9.8	68
Male	1.3	2.6	3.9	7.2	12.4	4.6	16.3	15.7	7.8	5.9	3.9	2.0	2.0	2.6	1.3	6.5	32
Total	6.5	17.0	22.9	22.2	19.0	12.4	50.3	49.7	23.5	17.0	12.4	13.1	9.2	3.9	4.6	16.3	100

AU, Adelaide University; CSU, Charles Sturt University; JCU, James Cook University; MU, Murdoch University; UM, Melbourne university; UQ, The University of Queensland; USYD, The University of Sydney.

### Analgesics prescribed

**Figure 1:**
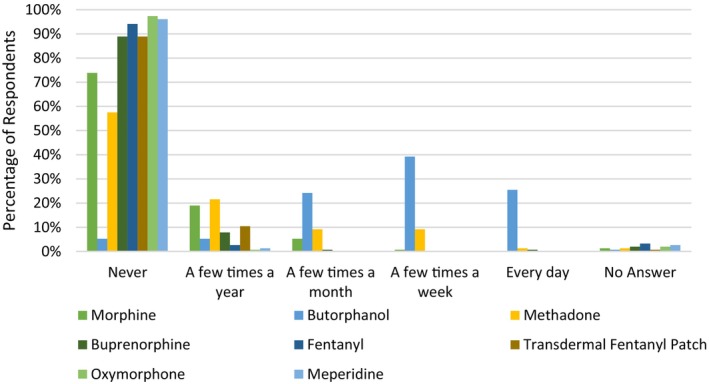
Frequency of opioid administration in horses by 153 Australian veterinarians.

**Figure 2:**
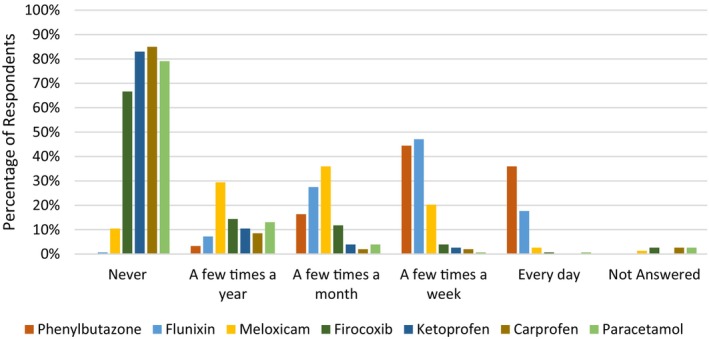
Frequency of nonsteroidal anti‐inflammatory (NSAID) administration in horses by 153 Australian veterinarians.

**Figure 3:**
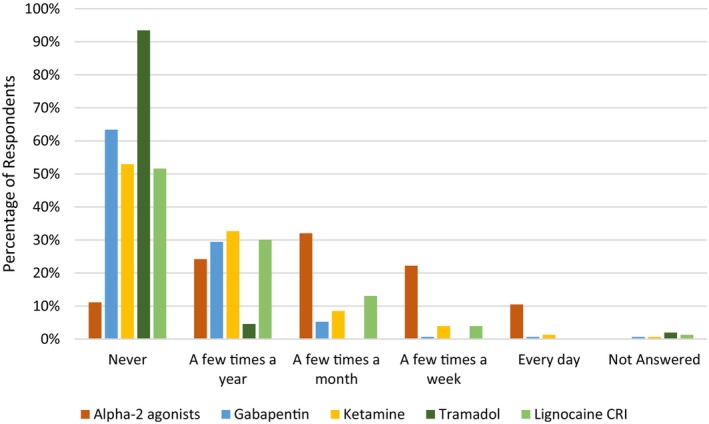
Frequency of nonopioid and non‐NSAID analgesic administration in horses by 153 Australian veterinarians.

**Figure 4:**
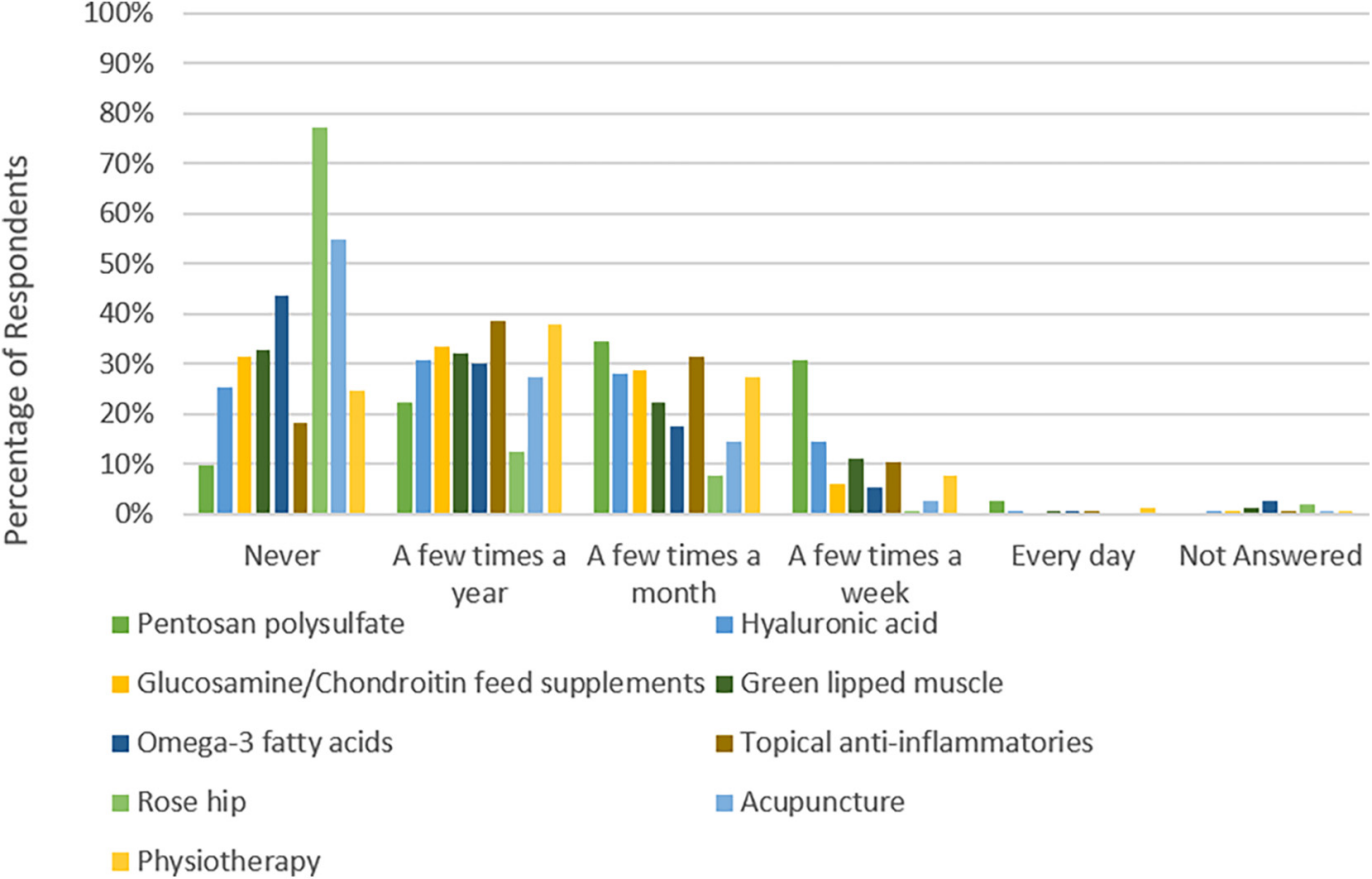
Frequency of adjunct analgesic therapy used in horses by 153 Australian veterinarians.

**Figure A1:**
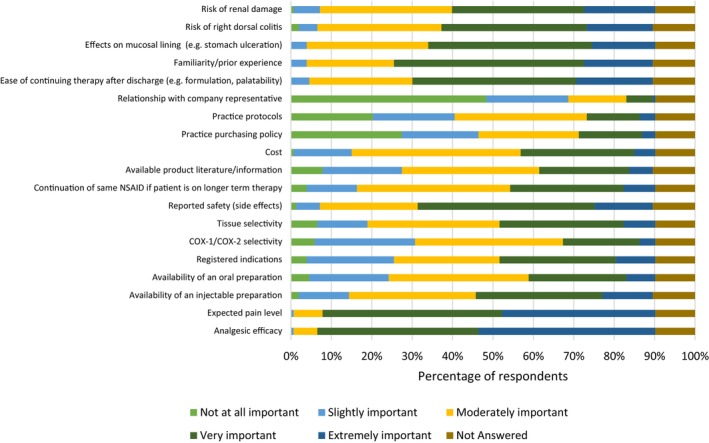
Factors influencing perioperative NSAID choice on a 5‐point scale. Scale ranging from 1 = “not at all important” to 5 = “extremely important”.

The most commonly administered opioid was butorphanol with 25.5% and 39.2% of respondents using it “every day” and “a few times a week”, respectively. All other opioids were used infrequently (Figure 1). The use of NSAIDs was more diverse with phenylbutazone, flunixin and meloxicam being the most commonly administered (Figure 2). Nonopioid and non‐NSAID analgesics were infrequently utilised by respondents with alpha2 agonists (excluding their use for the sole purpose of sedation) used most frequently (Figure 3). Adjunctive therapies were infrequently utilised by respondents (Figure 4). The most frequently utilised adjunctive therapy was pentosan polysulphate with 30.7% of respondents utilising it weekly. *Factors influencing perioperative NSAID choice can be found in* Figure A1.

### Use of analgesics in the Perioperative period and factors influencing medication selection

**Figure 5:**
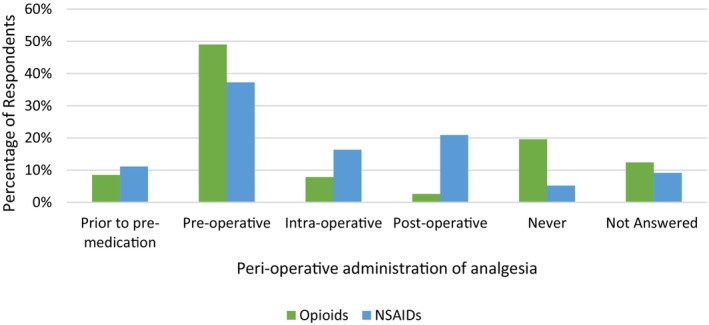
Perioperative administration of non steroidal anti‐inflammatory drugs (NSAIDs) and opioids to horses by 153 Australian veterinarians. Prior to premedication: Time from 12 h prior to surgery to immediately prior to routine premedication. Preoperative: Time of premedication to induction of anaesthesia. Intraoperative: Time from induction of anaesthesia to extubation/recovery (horse in sternal recumbency). Postoperative: Time from anaesthetic recovery (horse in sternal recumbency or standing) onward.

**Figure A2:**
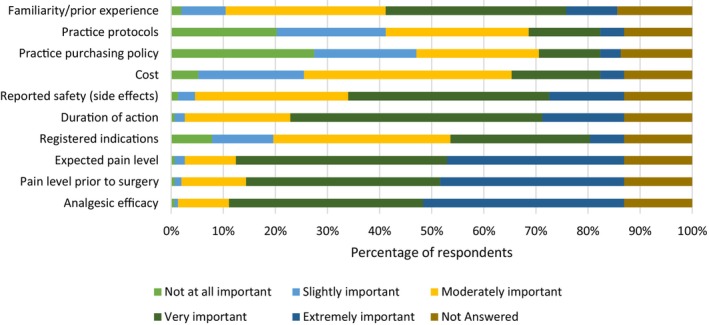
Factors influencing perioperative opioid choice, on a 5‐point scale. Scale ranging from “1 = not at all important” to “5 = extremely important”.

When considering the perioperative period, NSAIDs (37.3%) and opioids (49%) were the most commonly administered (Figure 5). One fifth of respondents (19.6%) reported never administering opioids in the perioperative period. When respondents were questioned on what factors influenced their choice of opioid, the three highest scoring responses were “analgesic efficacy”, “pain level before surgery” and “expected pain level” (Figure A2).

### Pain scoring of horses

**Figure 6:**
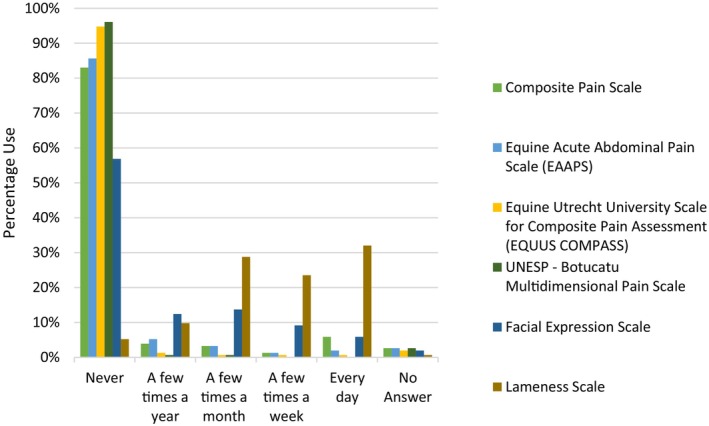
Frequency of pain scale use in horses by 153 Australian veterinarians.

Formal pain scales were infrequently utilised, with no pain scale being utilised commonly (daily or weekly) by more than 35% of respondents. Of pain scales utilised, the AAEP lameness scale was the most commonly used with 30.7% and 23.5% of respondents utilising this method daily and weekly, respectively (Figure 6). Of respondents who had access to a hospital, 98.9% (90/91) performed pain scoring. The pain scoring was performed by veterinarians 87.9% (80/91), by veterinary nurses 9.9% (9/91) and by veterinary students 1.1% (1/91) of the time; 1.1% of respondents report “no monitoring” when specifically asked who performs pain scoring.

### Perception of procedural pain in horses

**Figure 7:**
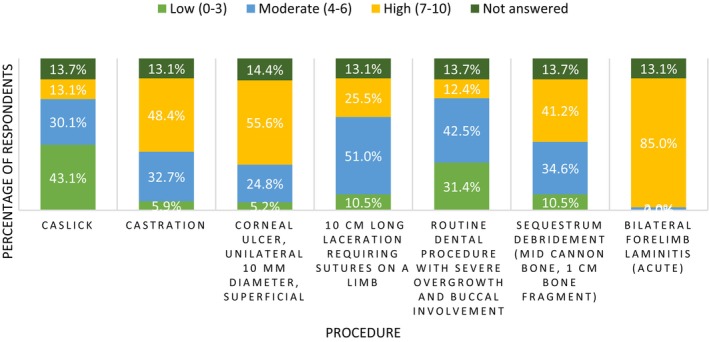
Perception of procedural pain in horses by 153 Australian Veterinarians, for seven different procedures, scored on an 11‐point scale (0–10) and assuming no prior analgesia provided.

**Figure B1:**
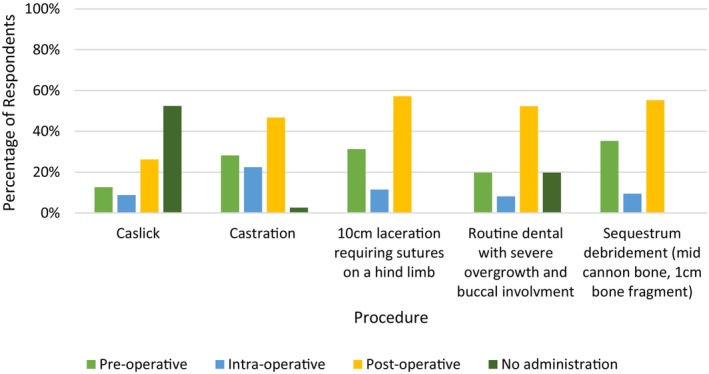
Timing of Non steroidal anti‐inflammatory administration to horses in the perioperative period by 153 Australian veterinarians.

Respondents were provided with seven clinical scenarios and asked to score these patient's perceived level of pain, within the first 24 h, on a 0–10 scale (0 being no pain, 10 being the worst pain imaginable). In these clinical and surgical scenarios respondents were asked to assume these patients had been provided with no analgesia. Figure 7 summarises these results. Respondents were then asked a series of questions in regard to the theoretical treatment of these conditions (more Appendix B and Figure B1) 0.4 Female respondents tended to designate higher perceived pain scores to all scenarios except “bilateral forelimb laminitis (acute)”. Trends in perception of procedural pain were consistent across respondents, independent of year of graduation.

**Figure 8:**
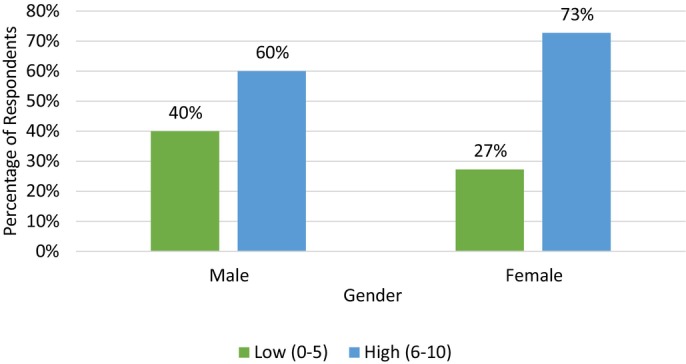
Average pain score of 153 Australian equine veterinarians categorised as low (0–5) or high (6–10) in relation to respondent gender.

**Figure 9:**
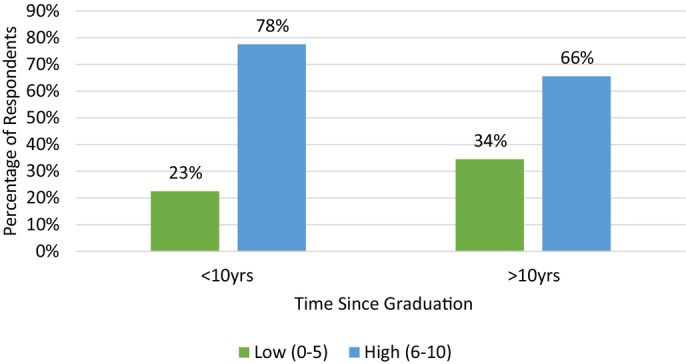
Average pain score of 153 Australian equine veterinarians categorised as low (0–5) or high (6–10) in relation to respondent time since graduation.

**Figure 10:**
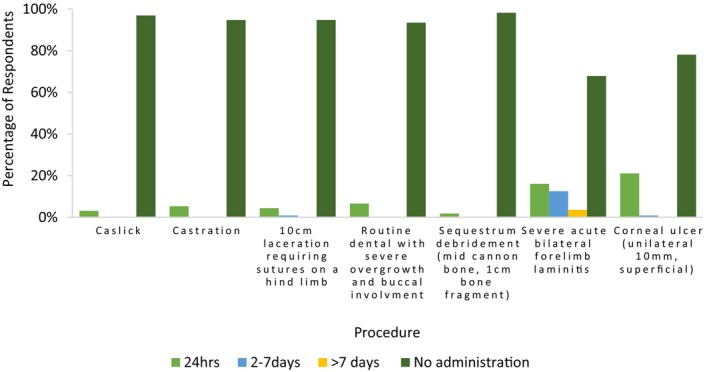
Duration of administration of opioids to horses for specific conditions by 153 Australian veterinarians, reported as percentage of respondents.

Females and those graduated less than 10 years were more likely to have a higher average pain score (Figures 8 and 9 respectively). Figure 10 explores ongoing opioid prescribing habits. Multivariable logistic regression of all data found the only variables to reach statistical significance (P < 0.05) were respondents who utilise opioids in the perioperative period for routine dentals and respondents who scored corneal ulceration a high pain score and were more likely to have been designated as a “High” average pain scorer (OR 2.64) (CI 1.05–6.64). All other variables did not reach significance.

## Discussion

The provision of analgesia is of the utmost importance in veterinary medicine. This survey found that equine veterinary practitioners in Australia frequently treat pain in horses, with NSAIDs and opioids, with 25% or more respondents administering these drugs every day. Respondents less frequently administered adjunctive medications outside of these categories. Veterinarians in this study infrequently used formal pain scales and were found to have a wide variation in their perceptions of pain.

Survey respondent demographics are different to previous international publications regarding equine analgesia, with a higher percentage of female respondents in our study.^27^, ^28^, ^29^ Differences between our findings and previous reports are likely due to geographic and temporal variations as our results mirror recently published Australian small animal and industry‐wide data.^30^ This is likely due to a higher proportion of female equine veterinarians; however, it may reflect a higher degree of female engagement with the survey.

Respondents reported opioids and NSAIDs as their most utilised analgesics in this study. While global comparison of the type and frequency of analgesic administration in horses is difficult to perform as few surveys have recorded frequency of use, the results of this survey appear consistent with other reports.^27^, ^28^ When examining the use of opioids in this survey, the kappa agonist and mu antagonist, butorphanol, was the most frequently administered which is a consistent finding across the literature.^27^, ^28^, ^29^ Butorphanol has historically been popular in equine medicine, being viewed as an effective visceral analgesic and being licensed for use in horses in most countries.^1^ This belief is largely related to early experimental studies suggesting butorphanol's analgesic potential; however, clinical data attesting to butorphanol's analgesic efficacy are lacking with few clinical studies demonstrating benefit and much contradictory data existing.^2^, ^8^, ^31^, ^32^, ^33^, ^34^, ^35^ In a 2019 clinical guideline on equine analgesia by the British Equine Veterinary Association stated that butorphanol has poor analgesic efficacy and that other opioid options, such as methadone or buprenorphine, should be utilised for the prevention and treatment of pain.^17^, ^36^ A potential explanation for this discrepancy between literature recommendations and current clinical practice is a lack of continuing education or awareness by practicing clinicians. Lack of access to other analgesic medications cannot be excluded as a potential cause as drug availability data were not collected in this survey. Use of full and partial mu opioid agonists, such as methadone or buprenorphine, was found to be far less common (Figure 1) and while evidence of mu agonist efficacy in equine patients is minimal and largely nonclinical, it is suggested they may provide more analgesia than other opioid options.^8^, ^17^, ^34^, ^37^, ^38^, ^39^ This disparity between literature and clinical practices demonstrates the need for robust clinical evidence and ongoing education. It must be acknowledged that improvements in Australian veterinarian education may have occurred since this survey was conducted and attitudes and practices in the current equine landscape may differ.

Veterinarians in this study were unlikely to prescribe ongoing opioids to horses. This is in contrast to small animal medicine where the prescription of opioids for a number of days is common place.^30^ The reason for this is likely multifactorial. Many of the reported conditions are seen on an ambulatory basis and the ongoing provision of opioids to these patients would likely require clinicians to dispense large volumes of opioids for owners to administer. In Australia, all opioids excluding the opioid‐like drug tramadol are schedule 8 controlled drugs. Potential for owner misuse may be grounds for a reluctance to prescribe. Side‐effect profiles may also be a factor in the reluctance of ongoing opioid prescription. Of respondents, 53% listed reported side effects as either “very important” or “extremely important” when choosing an opioid to prescribe. Opioids have been reported to result in ileus—potentially precipitating colic, and excitation in horses.^8^ Much of the evidence for these conclusions has arisen from the experimental administration of opioids to nonpainful horses and while evidence is spare, the risks of these side effects are likely lessened in a clinical setting.^17^ Practitioners must also appreciate the decrease in gastrointestinal motility that untreated pain may cause.^40^

When describing veterinary analgesic use it is important to contextualise these data by considering how respondents perceive pain and identify avenues of pain recognition. The current study illustrates how painful the survey respondents perceive certain procedures and conditions (Figure 7). Our findings in relation to respondent demographics and allocated pain score mirror that of previously published reports across both human and veterinary literature, with female respondents being more likely to score procedures as highly painful (Figure 8).^30^, ^41^ While an exact cause for this gender difference is unknown it is suspected that female clinicians may exhibit a greater degree of empathy; therefore, be more likely to consider procedures as highly painful.^42^ Similar conditions as those examined in our survey have been previously studied in two surveys—a 2010 survey of New Zealand veterinarians and a 2002 survey of veterinarians in the United Kingdom.^27^, ^28^ In the current study, respondents tended to rate procedures more painful than in previous reports. In the 2010 study, New Zealand veterinarians perceived all comparable conditions (Caslick's procedures, castrations and severe laminitis) as less painful than respondents of the current study.^28^ This may simply be the result of geographic attitude differences or may signify an increasing focus on pain by the veterinary community. The perioperative and postoperative provision of analgesia appears to trend logically. Conditions with higher perceived pain scores appear to have a greater provision of analgesia and are provided with longer durations of analgesia. This trend has been demonstrated previously in similar veterinary surveys regarding pain in dogs and cats.^30^, ^43^

A distinct lack of uniform pain scoring was also noted by survey respondents. Likely reasons for this include the lack of a validated pain scoring system for horses or a lack of education regarding the utility of pain scoring. Human patients presenting to the emergency room who received pain scoring were administered significantly more analgesia, and it was administered more promptly to those patients with high pain scores.^44^ In this study, the human emergency department retrospectively assessed analgesia administration before and after implementing mandatory pain scoring and found a greater prescription of analgesia after the implementation of pain scoring. Although no robust validated pain scales exist in equine medicine, the routine use of a standardised scoring system is recommended. This ensures the practitioner/s remains cognisant of the patients' pain routinely throughout the treatment period, adjusting for dynamic changes. This survey demonstrates the clear need for improvement in this area. The use of formal pain scoring was much higher in those respondents who had access to an equine hospital. Some hospitals, especially teaching hospitals, may have a requirement to use pain scoring on all patients. Hospitalised horses are likely to reflect more intensively managed cases, and hence those cases are more likely to be receiving tailored analgesic administration in response to variable perceived pain and hence the greater use of formal pain scoring within a hospital environment. Although many pain scores have poor interobserver repeatability, hospitalised horses are likely to be examined by numerous staff and students, and therefore use of a formal pain score can help with patient care and transfer between clinicians, students and nursing shifts.

A limitation of the current study is the inherent “black and white” nature of survey‐based data collection. This is especially apparent when collecting data regarding a subjective, dynamic field such as pain management. Selection bias may also skew results as this survey was self‐enrolled. Participants who completed the survey may be those with a special interest in pain management—skewing results in favour of optimal management. However, an attempt to ameliorate this effect was made by offering an incentive of a lucky draw for an iPad™ for survey completion.

## Conclusion

This survey finds that analgesia administration in Australian horses is largely limited to the use of butorphanol and NSAIDs. It also demonstrates that horses are infrequently pain scored and many veterinarians do not score common surgical procedures or conditions as highly painful. This is a clear rationale for continuing education and improving pain management by Australian veterinarians.

## Conflicts of interest and sources of funding

The authors declare no conflicts of interest for the work presented here. Funding for this study was provided by Boehringer Ingelheim Animal Health Australia.

## Supporting information


**Data S1:** Appendix

## Figures and Tables

**Figure B2:**
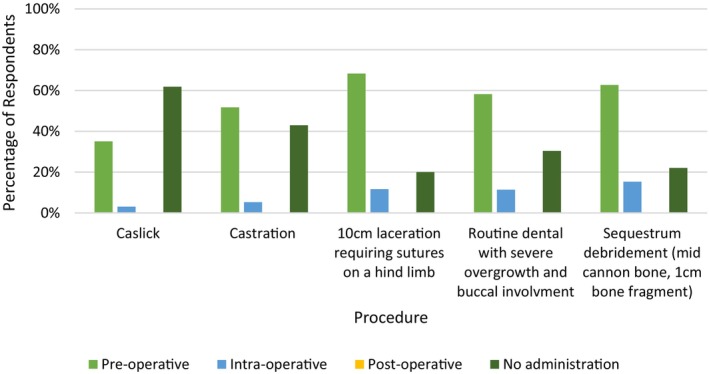
Timing of opioid administration to horses in the perioperative period by 153 Australian veterinarians.

**Figure B3:**
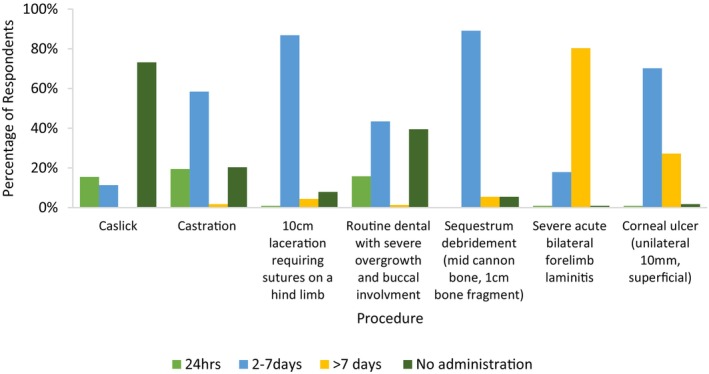
Duration of administration of NSAIDs to horses for specific conditions by 153 Australian veterinarians, reported as percentage of respondents.

## Data Availability

The data that support the findings of this study are available on request from the corresponding author. The data are not publicly available due to privacy or ethical restrictions.

## Appendix B Analgesia provision in relation to specific procedure or condition

Figures B1 and B2 demonstrate the timing of analgesic (NSAID and opioid) administration by respondents when considering specific conditions or procedures. Responses were not mutually exclusive, allowing respondents to elect multiple periods to administer analgesia. Figure B3 shows the duration of analgesic administration by respondents for these specific conditions.
