# Supplementary material for: Survey describing the perspectives and practices of Australian veterinarians to pain management in horses
Source: Aust Vet J. 2026 Feb 5;104(5):287–97. doi: 10.1111/avj.70059 (PMC13132014; doi:10.1111/avj.70059)
Supplement: Supplementary file 1 — Data S1: Appendix [file AVJ-104-287-s001.docx]

Equine Pain Management Survey

Survey Flow

Standard: Introduction (1 Question)

Block: Demographics (11 Questions)

Standard: Pharmacy Questions (5 Questions)

Standard: Pain Monitoring (3 Questions)

Standard: General Pain Management Questions (7 Questions)

Standard: Block 1 (1 Question)

Standard: Hospital procedures (11 Questions)

Standard: Ambulatory Procedures (16 Questions)

Standard: Pain assessment in adult horses (1 Question)

Standard: OA - medical (8 Questions)

Standard: Conclusion (1 Question)

Branch: New Branch

If

If Would you like to enter the competition (will need to provide name and email address - data enter... Yes Is Selected

EndSurvey: Advanced

Branch: New Branch

If

If Would you like to enter the competition (will need to provide name and email address - data enter... No Is Selected

EndSurvey:

| Page Break |  |
| --- | --- |

Start of Block: Introduction

Q1
**Equine Pain Management Survey**

 Thank you for your interest in this survey. Boehringer Ingelheim Veterinary Medical Services, in conjunction with veterinarians from the University of Queensland and Randwick Equine Centre, have developed this pain management survey to investigate the attitudes and practices of veterinarians in Australia to acute and chronic pain management in horses.  By taking part in this first of its kind research you can add your voice to that of the profession and help us advance the management and wellbeing of our equine patients.

 The survey takes 15-20 minutes to complete and is best taken on larger device (laptop, tablet etc.).  All responses are anonymous.  If you would like to enter the competition you will be required to enter your contact details, however these details are not linked to your survey answers.

 Thank you for your taking part in this exciting research! 
 

End of Block: Introduction

Start of Block: Demographics

| 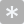 |
| --- |

Q2 In what year did you graduate from veterinary school/university?

________________________________________________________________

Q3 Which veterinary school/university did you graduate  from?

- University of Sydney
- University of Melbourne
- University of Queensland
- Murdoch University
- University of Adelaide
- James Cook University
- Charles Sturt University
- Massey University
- Other (please enter below) ________________________________________________

Q4 Have you completed an internship?

- Yes
- No

Q5 Have you completed a residency?

- Yes
- No

Q6
Are you a registered specialist?

- Yes
- No

Skip To: Q8 If Are you a registered specialist? = No

Q7
In which areas are you a registered specialist? (select all that apply)

- Animal Behaviour
- Equine Medicine
- Equine Surgery
- Large Animal Medicine
- Large Animal Surgery
- Veterinary Anaesthesia
- Veterinary Anaesthesia and Analgesia
- Veterinary Emergency Medicine and Critical Care
- Veterinary Ophthalmology
- Veterinary Reproduction
- Other ________________________________________________

Q8 What is your gender?

- Male
- Female
- Prefer not to answer

| 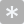 |
| --- |

Q9 In which postcode is your primary place of work located?

________________________________________________________________

Q10 In a typical working week what percentage of your cases are equine patients?

- 100%
- 75-99%
- 50-74%
- 10-49%
- None

Skip To: End of Survey If In a typical working week what percentage of your cases are equine patients? = None

Q11 Please indicate how frequently you typically see the following types of horses in practice.

|  | Never | A few times a year | A few times a month | A few times a week | Every day |
| --- | --- | --- | --- | --- | --- |
| Thoroughbred race horses (currently racing) |  |  |  |  |  |
| Standardbred race horses (currently racing) |  |  |  |  |  |
| Race horses that are spelling |  |  |  |  |  |
| Sport or competition horses (not racing) |  |  |  |  |  |
| Breeding stock |  |  |  |  |  |
| Pleasure horse |  |  |  |  |  |

Q12 What type of practice do you work in?

- Full service equine only hospital (with internal medicine and surgical facilities)
- Full service equine hospital (with internal medicine and surgical facilities) within a mixed animal hospital
- Ambulatory, but based out of an equine hospital
- Ambulatory, but based out of a mixed animal hospital
- Ambulatory only, no associated hospital

End of Block: Demographics

Start of Block: Pharmacy Questions

Q13 Please indicate how frequently you prescribe (either being used in hospital or for owner administration) the following NSAIDs to individual equine cases at your practice.

|  | Never | A few times a year | A few times a month | A few times a week | Every day |
| --- | --- | --- | --- | --- | --- |
| Phenylbutazone |  |  |  |  |  |
| Flunixin |  |  |  |  |  |
| Meloxicam |  |  |  |  |  |
| Firocoxib |  |  |  |  |  |
| Ketoprofen |  |  |  |  |  |
| Carprofen |  |  |  |  |  |
| Paracetamol |  |  |  |  |  |

Q14 Please indicate how frequently you use or prescribe the following opioids to individual equine cases at your practice.

|  | Never | A few times a year | A few times a month | A few times a week | Every day |
| --- | --- | --- | --- | --- | --- |
| Buprenorphine |  |  |  |  |  |
| Butorphanol |  |  |  |  |  |
| Fentanyl inection |  |  |  |  |  |
| Fentanyl transdermal patch |  |  |  |  |  |
| Methadone |  |  |  |  |  |
| Morphine |  |  |  |  |  |

Q15 Please indicate how frequently you use or prescribe the following local anaesthetics for providing analgesia (excluding for diagnostic nerve blocks) to individual equine cases at your practice. 

|  | Never | A few times a year | A few times a month | A few times a week | Every day |
| --- | --- | --- | --- | --- | --- |
| Lignocaine/lidocaine |  |  |  |  |  |
| Bupivacaine |  |  |  |  |  |
| Mepivacaine |  |  |  |  |  |
| Prilocaine |  |  |  |  |  |
| Proxymetacaine |  |  |  |  |  |
| Ropivacaine |  |  |  |  |  |

Q16 Please indicate how frequently you use or prescribe the following drugs as **analgesics** in horses at your practice.  (exclude instances when drugs used for non-analgesic effects)

|  | Never | A few times a year | A few times a month | A few times a week | Every day |
| --- | --- | --- | --- | --- | --- |
| alpha-2 agonists |  |  |  |  |  |
| Gabapentin |  |  |  |  |  |
| Ketamine (excluding for general anaesthesia) |  |  |  |  |  |
| Tramadol |  |  |  |  |  |
| Oxymorphone |  |  |  |  |  |
| Meperidine |  |  |  |  |  |
| Lidocaine CRI |  |  |  |  |  |

Q17 Please indicate how frequently you use or recommend the following adjunct or non-traditional treatments in horses at your practice.

|  | Never | A few times a year | A few times a month | A few times a week | Every day |
| --- | --- | --- | --- | --- | --- |
| Pentosan Polysulfate |  |  |  |  |  |
| Hyaluronic acid |  |  |  |  |  |
| Glucosamine/Chondroitin feed supplements |  |  |  |  |  |
| Green Lipped Mussel products |  |  |  |  |  |
| Omega 3s |  |  |  |  |  |
| Topical anti-inflammatory |  |  |  |  |  |
| Rose Hip |  |  |  |  |  |

End of Block: Pharmacy Questions

Start of Block: Pain Monitoring

Q18 Please indicate how often you have used the following pain scales

|  | Never | A few times a year | A few times a month | A few times a week | Every day |
| --- | --- | --- | --- | --- | --- |
| CPS (composite pain scale) |  |  |  |  |  |
| EAAPS (equine acute abdominal pain scale) |  |  |  |  |  |
| EQUUS-COMPASS (Equine Utrecht University Scale for Composite Pain Assessment) |  |  |  |  |  |
| UNESP-Botucatu multidimensional pain scale |  |  |  |  |  |
| Facial Expression Scale |  |  |  |  |  |
| Lameness Scale |  |  |  |  |  |

Q19 In an equine hospital setting who is primarily responsible for evaluating a patient’s pain levels? (If you don’t work in a hospital setting select N/A)

- Veterinarian
- Veterinary intern
- Veterinary nurse
- Veterinary student
- No monitoring
- N/A (I do not work in hospital setting)

Q20
Following a routine ambulatory procedure (such as a caslick or castration), who is most commonly evaluating a patient’s pain level? (if you do not do ambulatory procedures select N/A)

- Owner
- Trainer
- Stable staff
- Veterinarian
- No monitoring
- N/A

End of Block: Pain Monitoring

Start of Block: General Pain Management Questions

Q21
The following questions relate to your routine (>80% of the time) use of NSAIDs and opioids in the peri-operative period for horses in a hospital setting, pertaining to surgeries performed under general anaesthesia. Consider the following definitions.
Prior to premedication:  Time from 12 hours prior to surgery to time of routine pre-medication. 

Pre-operative: Time from premedication to induction of anaesthesia.
Intra-operative: Time from induction of anaesthesia to extubation/recovery (horse in sternal recumbency).
Post-operative: Time from anaethetic recovery (horse in sternal recumbancy or standing) onward.

Q22 In the peri-operative period, in  a pain free healthy horse when do you first administer the NSAID and/or opioids?

|  | Never | Prior to pre-medication | Pre-operative | Intra-operative | Post-operative |
| --- | --- | --- | --- | --- | --- |
| NSAID |  |  |  |  |  |
| Opioid |  |  |  |  |  |

Q23 When using NSAIDs pre- and/or intra-operatively which of the following tests/monitoring do you routinely perform (> then 80% of the time)?  If you routinely perform these only in some patient classes, please indicate which ones.

|  | **Routinely perform** | | | **Patient class** | | | | | |
| --- | --- | --- | --- | --- | --- | --- | --- | --- | --- |
|  | No | Yes, some horses | Yes, all horses | Healthy foal | Healthy foal (1 week to 1 year) | Healthy horse (1 to 10 years) | Healthy horse (11 to 20 years) | Healthy horse (> 20 year) | Critically ill patient (any age) |
| Complete blood count |  |  |  |  |  |  |  |  |  |
| Biochemistry |  |  |  |  |  |  |  |  |  |
| Fibrinogen |  |  |  |  |  |  |  |  |  |
| White blood cell count (only) |  |  |  |  |  |  |  |  |  |
| Urinalysis |  |  |  |  |  |  |  |  |  |
| IV Fluid Therapy |  |  |  |  |  |  |  |  |  |
| Blood Pressure Monitoring |  |  |  |  |  |  |  |  |  |

Q24 When considering which NSAID to give in the peri-operative/post-operative period to a horse how important do you rate the following factors?

|  | Not at all important | Slightly important | Moderately important | Very important | Extremely important |
| --- | --- | --- | --- | --- | --- |
| Analgesic efficacy |  |  |  |  |  |
| Expected pain level |  |  |  |  |  |
| Availability of an injectable preparation |  |  |  |  |  |
| Availability of an oral preparation |  |  |  |  |  |
| Registered indications |  |  |  |  |  |
| COX1/COX 2 selectivity |  |  |  |  |  |
| Tissue selectivity |  |  |  |  |  |
| Reported safety (side effects) |  |  |  |  |  |
| Continuation of same NSAID if patient is on longer term therapy |  |  |  |  |  |
| Available product literature/information |  |  |  |  |  |
| Cost |  |  |  |  |  |
| Practice Purchasing policy |  |  |  |  |  |
| Practice Protocols |  |  |  |  |  |
| Relationship with company representative |  |  |  |  |  |
| Ease of continuing therapy after discharge (eg formulation, palatability |  |  |  |  |  |
| Familiarity/prior experience |  |  |  |  |  |
| Effects on mucosal lining (eg stomach ulceration) |  |  |  |  |  |
| Right dorsal colitis |  |  |  |  |  |
| Renal damage |  |  |  |  |  |

Q25 When considering which opioid to give in the peri-operative/post-operative period to a horse how important do you rate the following factors?

|  | Not at all important | Slightly important | Moderately important | Very important | Extremely important |
| --- | --- | --- | --- | --- | --- |
| Analgesic efficacy |  |  |  |  |  |
| How painful the animal is prior to surgery |  |  |  |  |  |
| Expected pain level during surgery |  |  |  |  |  |
| Registered indications |  |  |  |  |  |
| Duration of action |  |  |  |  |  |
| Tissue selectivity |  |  |  |  |  |
| Reported safety (side effects) |  |  |  |  |  |
| Cost |  |  |  |  |  |
| Practice Purchasing policy |  |  |  |  |  |
| Practice Protocols |  |  |  |  |  |
| Familiarity/prior experience |  |  |  |  |  |
| Expected pain level |  |  |  |  |  |

Q26 Do you  use any local anaesthetic (LA) techniques in horses to facilitate procedures (not for diagnostics)?

- Yes
- No

Skip To: End of Block If Do you use any local anaesthetic (LA) techniques in horses to facilitate procedures (not for diag... = No

Q27 If yes, please select which ones you routinely use (routinely used defined as being used in greater than 80% cases where such use would be indicated).

- Distal limb blocks (high 4 point and below)
- Proximal limb blocks
- Intratesticular local for castrations
- Maxillary and mandibular blocks for dental procedures
- Local infiltration for dentals
- Periocular local blocks for ocular surgery , retrobulbar)
- Splash blocks for soft tissue
- C2/3 block for tieback surgeries
- Epidurals

End of Block: General Pain Management Questions

Start of Block: Block 1

Q28 The following questions relate to your use of drugs for providing analgesia to horses.
 There are two sections.  Section 1 relates to hospital based surgical procedures while section 2 relates to ambulatory procedures and medical conditions. Please indicate which drug(s) you would administer for the procedures, and for how long you would administer them post procedure. For medical conditions indicate the duration of treatment with each drug class.

 For each analgesic option select when you would use it (pre-op, intra-op, or post-op).  If you use a drug in more than one period, select all that are appropriate (e.g. if you give a pre-op dose of NSAID followed by ongoing post-op treatment, select the boxes for both pre-op and post-op).  For any analgesic used in the post-op period select the duration for which it is used. 

 If you do not use an analgesic option, leave it blank.

 **Answer example**: If you give an opioid, NSAID, and local anaesthetic in the pre-operative period, and continue patient on NSAID for 2 to 3 days post operatively, you should the complete the question as below.

End of Block: Block 1

Start of Block: Hospital procedures

Q29
The following section refers to hospital based surgical procedures.  
Do you perform hospital based surgical procedures?

- Yes
- No

Skip To: End of Block If The following section refers to hospital based surgical procedures.   Do you perform hospital bas... = No

Q30
**Exploratory celiotomy for a large colon volvulus.**
Do you perform this procedure?

- Yes
- No

Skip To: Q32 If Exploratory celiotomy for a large colon volvulus.  Do you perform this procedure? = No

Q31
**Exploratory celiotomy for a large colon volvulus.**
If used, when would you give each class of analgesic drug?  If used post-operatively, please provide duration of treatment.

|  | **Use** | | | **Duration post-op** | | | | |
| --- | --- | --- | --- | --- | --- | --- | --- | --- |
|  | Pre-op | Intra-op | Post-op | 24 hours | 2-3 days | 4-5 days | 6-7 days | > 7 days |
| NSAID |  |  |  |  |  |  |  |  |
| Opioid |  |  |  |  |  |  |  |  |
| Local anaesthetic (non-CRI) |  |  |  |  |  |  |  |  |
| Alpha-2 agonist |  |  |  |  |  |  |  |  |
| Other |  |  |  |  |  |  |  |  |

| Page Break |  |
| --- | --- |

Q32
**Tie-back surgery.**
Do you perform this procedure?

- Yes
- No

Skip To: Q34 If Tie-back surgery.  Do you perform this procedure? = No

Q33
**Tie-back surgery.**
If used, when would you give each class of analgesic drug?  If used post-operatively, please provide duration of treatment.

|  | **Use** | | | **Duration post-op** | | | | |
| --- | --- | --- | --- | --- | --- | --- | --- | --- |
|  | Pre-op | Intra-op | Post-op | 24 hours | 2-3 days | 4-5 days | 6-7 days | > 7 days |
| NSAID |  |  |  |  |  |  |  |  |
| Opioid |  |  |  |  |  |  |  |  |
| Local anaesthetic (non-CRI) |  |  |  |  |  |  |  |  |
| Alpha-2 agonist |  |  |  |  |  |  |  |  |
| Other |  |  |  |  |  |  |  |  |

| Page Break |  |
| --- | --- |

Q34
**Surgical repair of deep corneal ulcer including debridement and placement of sub palpebral lavage.**
Do you perform this procedure?

- Yes
- No

Skip To: Q36 If Surgical repair of deep corneal ulcer including debridement and placement of sub palpebral lavage... = No

Q35
**Surgical repair of deep corneal ulcer including debridement and placement of sub palpebral lavage.**
If used, when would you give each class of analgesic drug?  If used post-operatively, please provide duration of treatment.

|  | **Use** | | | **Duration post-op** | | | | |
| --- | --- | --- | --- | --- | --- | --- | --- | --- |
|  | Pre-op | Intra-op | Post-op | 24 hours | 2-3 days | 4-5 days | 6-7 days | > 7 days |
| NSAID |  |  |  |  |  |  |  |  |
| Opioid |  |  |  |  |  |  |  |  |
| Local anaesthetic (non-CRI) |  |  |  |  |  |  |  |  |
| Alpha-2 agonist |  |  |  |  |  |  |  |  |
| Other |  |  |  |  |  |  |  |  |

| Page Break |  |
| --- | --- |

Q36
**Condylar fracture of the third metacarpal (cannon) bone.**
Do you perform this procedure?

- Yes
- No

Skip To: Q38 If Condylar fracture of the third metacarpal (cannon) bone.  Do you perform this procedure? = No

Q37
**Condylar fracture of the third metacarpal (cannon) bone.**
If used, when would you give each class of analgesic drug?  If used post-operatively, please provide duration of treatment.

|  | **Use** | | | **Duration post-op** | | | | |
| --- | --- | --- | --- | --- | --- | --- | --- | --- |
|  | Pre-op | Intra-op | Post-op | 24 hours | 2-3 days | 4-5 days | 6-7 days | > 7 days |
| NSAID |  |  |  |  |  |  |  |  |
| Opioid |  |  |  |  |  |  |  |  |
| Local anaesthetic (non-CRI) |  |  |  |  |  |  |  |  |
| Alpha-2 agonist |  |  |  |  |  |  |  |  |
| Other |  |  |  |  |  |  |  |  |

| Page Break |  |
| --- | --- |

Q38
**Bilateral fetlock arthroscopy.**
Do you perform this procedure?

- Yes
- No

Skip To: End of Block If Bilateral fetlock arthroscopy.   Do you perform this procedure? = No

Q39
Bilateral fetlock arthroscopy**.**
If used, when would you give each class of analgesic drug?  If used post-operatively, please provide duration of treatment.

|  | **Use** | | | **Duration post-op** | | | | |
| --- | --- | --- | --- | --- | --- | --- | --- | --- |
|  | Pre-op | Intra-op | Post-op | 24 hours | 2-3 days | 4-5 days | 6-7 days | > 7 days |
| NSAID |  |  |  |  |  |  |  |  |
| Opioid |  |  |  |  |  |  |  |  |
| Local anaesthetic (non-CRI) |  |  |  |  |  |  |  |  |
| Alpha-2 agonist |  |  |  |  |  |  |  |  |
| Other |  |  |  |  |  |  |  |  |

| Page Break |  |
| --- | --- |

End of Block: Hospital procedures

Start of Block: Ambulatory Procedures

Q40
The following section refers to ambulatory practice based surgical procedures or conditions managed in ambulatory practice.  
Do you perform ambulatory practice?

- Yes
- No

Skip To: End of Block If The following section refers to ambulatory practice based surgical procedures or conditions manag... = No

Q41
**Castration.**
Do you perform this procedure?

- Yes
- No

Skip To: Q44 If Castration. Do you perform this procedure? = No

Q42 What type of castration do you perform?

- Standing castration using standard emasculator technique
- Castration in dorsal or lateral recumbency using standard emasculator technique
- Castration in dorsal or lateral recumbency using the Henderson gelding technique

| Page Break |  |
| --- | --- |

Q43
**${Q42/ChoiceGroup/SelectedChoices}.**
If used, when would you give each class of analgesic drug?  If used post-operatively, please provide duration of treatment.

|  | **Use** | | | **Duration post-op** | | | | |
| --- | --- | --- | --- | --- | --- | --- | --- | --- |
|  | Pre-op | Intra-op | Post-op | 24 hours | 2-3 days | 4-5 days | 6-7 days | > 7 days |
| NSAID |  |  |  |  |  |  |  |  |
| Opioid |  |  |  |  |  |  |  |  |
| Local anaesthetic (non-CRI) |  |  |  |  |  |  |  |  |
| Alpha-2 agonist |  |  |  |  |  |  |  |  |
| Other |  |  |  |  |  |  |  |  |

| Page Break |  |
| --- | --- |

Q44
**Routine Dental procedure with severe overgrowth and buccal involvement.**
Do you perform this procedure?

- Yes
- No

Skip To: Q46 If Routine Dental procedure with severe overgrowth and buccal involvement.  Do you perform this proc... = No

Q45
**Routine Dental procedure with severe overgrowth and buccal involvement.**
If used, when would you give each class of analgesic drug?  If used post-operatively, please provide duration of treatment.

|  | **Use** | | | **Duration post-op** | | | | |
| --- | --- | --- | --- | --- | --- | --- | --- | --- |
|  | Pre-op | Intra-op | Post-op | 24 hours | 2-3 days | 4-5 days | 6-7 days | > 7 days |
| NSAID |  |  |  |  |  |  |  |  |
| Opioid |  |  |  |  |  |  |  |  |
| Local anaesthetic (non-CRI) |  |  |  |  |  |  |  |  |
| Alpha-2 agonist |  |  |  |  |  |  |  |  |
| Other |  |  |  |  |  |  |  |  |

| Page Break |  |
| --- | --- |

Q46
**Caslicks.**
Do you perform this procedure?

- Yes
- No

Skip To: Q48 If Caslicks.  Do you perform this procedure? = No

Q47
**Caslicks.**
If used, when would you give each class of analgesic drug?  If used post-operatively, please provide duration of treatment.

|  | **Use** | | | **Duration post-op** | | | | |
| --- | --- | --- | --- | --- | --- | --- | --- | --- |
|  | Pre-op | Intra-op | Post-op | 24 hours | 2-3 days | 4-5 days | 6-7 days | > 7 days |
| NSAID |  |  |  |  |  |  |  |  |
| Opioid |  |  |  |  |  |  |  |  |
| Local anaesthetic (non-CRI) |  |  |  |  |  |  |  |  |
| Alpha-2 agonist |  |  |  |  |  |  |  |  |
| Other |  |  |  |  |  |  |  |  |

| Page Break |  |
| --- | --- |

Q48
**10 cm long laceration requiring sutures on a hind limb.**
Do you perform this procedure?

- Yes
- No

Skip To: Q50 If 10 cm long laceration requiring sutures on a hind limb.  Do you perform this procedure? = No

Q49
**10 cm long laceration requiring sutures on a hind limb.**
If used, when would you give each class of analgesic drug?  If used post-operatively, please provide duration of treatment.

|  | **Use** | | | **Duration post-op** | | | | |
| --- | --- | --- | --- | --- | --- | --- | --- | --- |
|  | Pre-op | Intra-op | Post-op | 24 hours | 2-3 days | 4-5 days | 6-7 days | > 7 days |
| NSAID |  |  |  |  |  |  |  |  |
| Opioid |  |  |  |  |  |  |  |  |
| Local anaesthetic (non-CRI) |  |  |  |  |  |  |  |  |
| Alpha-2 agonist |  |  |  |  |  |  |  |  |
| Other |  |  |  |  |  |  |  |  |

| Page Break |  |
| --- | --- |

| Page Break |  |
| --- | --- |

Q50
**Sequestrum debridement (mid cannon bone, 1 cm bone fragment).**
Do you perform this procedure?

- Yes
- No

Skip To: Q52 If Sequestrum debridement (mid cannon bone, 1 cm bone fragment).  Do you perform this procedure? = No

Q51
**Sequestrum debridement (**mid cannon bone, 1 cm bone fragment**).**
If used, when would you give each class of analgesic drug?  If used post-operatively, please provide duration of treatment.

|  | **Use** | | | **Duration post-op** | | | | |
| --- | --- | --- | --- | --- | --- | --- | --- | --- |
|  | Pre-op | Intra-op | Post-op | 24 hours | 2-3 days | 4-5 days | 6-7 days | > 7 days |
| NSAID |  |  |  |  |  |  |  |  |
| Opioid |  |  |  |  |  |  |  |  |
| Local anaesthetic (non-CRI) |  |  |  |  |  |  |  |  |
| Alpha-2 agonist |  |  |  |  |  |  |  |  |
| Other |  |  |  |  |  |  |  |  |

| Page Break |  |
| --- | --- |

Q52
**Corneal ulcer (unilateral 10 mm diameter, superficial).**
Do you manage this condition in practice?

- Yes
- No

Skip To: Q54 If Corneal ulcer (unilateral 10 mm diameter, superficial).  Do you manage this condition in practice? = No

Q53
**Corneal ulcer (**unilateral 10 mm diameter, superficial)**.**
If used, for how long would you give each class of drug.  (leave row blank if not used)

|  | **Duration of treatment** | | | | |
| --- | --- | --- | --- | --- | --- |
|  | 24 hours | 2-3 days | 4-5 days | 6-7 days | > 7 days |
| NSAID |  |  |  |  |  |
| Opioid |  |  |  |  |  |
| Local anaesthetic (non-CRI) |  |  |  |  |  |
| Alpha-2 agonist |  |  |  |  |  |
| Other |  |  |  |  |  |

| Page Break |  |
| --- | --- |

Q54
**Condition: Severe acute bilateral forelimb laminitis.**
Do you manage this condition in practice?

- Yes
- No

Skip To: End of Block If Condition: Severe acute bilateral forelimb laminitis.  Do you manage this condition in practice? = No

Q55
**Condition: Severe acute bilateral forelimb laminitis.**
If used, for how long would you give each class of drug.  (leave row blank if not used)

|  | **Duration of treatment** | | | | |
| --- | --- | --- | --- | --- | --- |
|  | 24 hours | 2-3 days | 4-5 days | 6-7 days | > 7 days |
| NSAID |  |  |  |  |  |
| Opioid |  |  |  |  |  |
| Local anaesthetic (non-CRI) |  |  |  |  |  |
| Alpha-2 agonist |  |  |  |  |  |
| Other |  |  |  |  |  |

End of Block: Ambulatory Procedures

Start of Block: Pain assessment in adult horses

Q56 In your opinion how severe would the pain be in an adult horse undergoing the following procedures or diagnosed with the following diseases within the first 24 hours after procedure/diagnosis was conducted? Assume that NO analgesic drug was given for the following situations. Estimate pain severity on a 11-point scale where 0 is no pain and 10 is worst pain imaginable.

|  | 0 | 1 | 2 | 3 | 4 | 5 | 6 | 7 | 8 | 9 | 10 |
| --- | --- | --- | --- | --- | --- | --- | --- | --- | --- | --- | --- |
| Caslick |  |  |  |  |  |  |  |  |  |  |  |
| Castration |  |  |  |  |  |  |  |  |  |  |  |
| Corneal ulcer, unilateral 10 mm diameter, superficial |  |  |  |  |  |  |  |  |  |  |  |
| 10 cm long laceration requiring sutures on a limb |  |  |  |  |  |  |  |  |  |  |  |
| Routine dental procedure with severe overgrowth and buccal involvement |  |  |  |  |  |  |  |  |  |  |  |
| Sequestrum debridement (mid cannon bone, 1 cm bone fragment) |  |  |  |  |  |  |  |  |  |  |  |
| Bilateral forelimb laminitis (acute) |  |  |  |  |  |  |  |  |  |  |  |

End of Block: Pain assessment in adult horses

Start of Block: OA - medical

Q57 For the medical management of osteoarthritis, at what stage (mild/moderate/severe) do you typically recommend commencing the following treatments?  If you do not recommend a particular treatment select N/A.

|  | N/A | Mild osteoarthritis | Moderate osteoarthritis | Severe osteoarthritis |
| --- | --- | --- | --- | --- |
| NSAIDs |  |  |  |  |
| Pentosan Polysulfate Injections |  |  |  |  |
| Nutraceuticals - Glucosamine\Chondroitin formulations |  |  |  |  |
| Dietary supplements |  |  |  |  |
| Lifestyle Management (e.g. exercise restriction, weight management) |  |  |  |  |
| Long lasting intra-articular injections (ie stem cells ) |  |  |  |  |
| Acupuncture |  |  |  |  |
| Homeopathy |  |  |  |  |

Q58 When considering which NSAID to give to horses with osteoarthritis how important do you rate the following factors?

|  | Not at all important | Slightly important | Moderately important | Very important | Extremely important |
| --- | --- | --- | --- | --- | --- |
| Analgesic efficacy |  |  |  |  |  |
| Availability of an injectable preparation |  |  |  |  |  |
| Availability of an oral preparation |  |  |  |  |  |
| Registered indications |  |  |  |  |  |
| COX 1/COX 2 selectivity |  |  |  |  |  |
| Tissue selectivity |  |  |  |  |  |
| Reported safety (side effects) |  |  |  |  |  |
| Ease of administration for long term therapy (e.g. formulation, palatability) |  |  |  |  |  |
| Available product literature/information |  |  |  |  |  |
| Cost |  |  |  |  |  |
| Practice purchasing policy |  |  |  |  |  |
| Practice protocols |  |  |  |  |  |
| Relationship with company representative |  |  |  |  |  |
| Effects on mucosal lining (e.g. stomach ulceration) |  |  |  |  |  |
| Risk of right dorsal colitis |  |  |  |  |  |
| Risk of renal failure |  |  |  |  |  |
| Familiarity |  |  |  |  |  |

Q59 When considering which nutraceutical to give to horses with osteoarthritis how important do you rate the following factors?

|  | Not at all important | Slightly important | Moderately important | Very important | Extremely important |
| --- | --- | --- | --- | --- | --- |
| Published peer-reviewed evidence |  |  |  |  |  |
| Registered indications |  |  |  |  |  |
| Reported safety (side effects) |  |  |  |  |  |
| Ease of administration for long term therapy (e.g. formulation, palatability) |  |  |  |  |  |
| Available product literature/information |  |  |  |  |  |
| Cost |  |  |  |  |  |
| Practice purchasing policy |  |  |  |  |  |
| Practice protocols |  |  |  |  |  |
| Familiarity |  |  |  |  |  |

Q60 When prescribing NSAIDs for a horse with osteoarthritis for the first time, do you recommend routine screening blood tests?

- Never
- Yes, always
- Yes, but only if suspicious of pre-existing disease
- Yes, only in geriatric

Q61 When prescribing NSAIDs for a horse with osteoarthritis, do you recommend a proton pump inhibitor (e.g. omeprazole)?

- Never
- Yes, always
- Yes, but only if suspicious of pre-existing disease
- Yes, only in geriatric

Q62 For an otherwise healthy horse, how often do you recommend rechecks for horses prescribed a NSAID for long-term management of **osteoarthritis**?

- Never
- Monthly
- Every 3 months
- Every 6 months
- Every 12 months

Q63 When considering which NSAID to give horses with laminitis, how important is the effect the NSAID has on mucosal ulceration of gastrointestinal tract in your choice of drug?

- Extremely important
- Very important
- Moderately important
- Slightly important
- Not at all important

Q64 For an otherwise healthy horse, how often do you recommend rechecks for horses prescribed a NSAID for long-term management of **laminitis**?

- Never
- Monthly
- Every 3 months
- Every 6 months
- Every 12 months

End of Block: OA - medical

Start of Block: Conclusion

Q65 Would you like to enter the competition (will need to provide name and email address - data entered in the survey questions remains anonymous)?

- Yes
- No

End of Block: Conclusion
